# Mandibular dental implant placement immediately after teeth removal in head and neck cancer patients

**DOI:** 10.1007/s00520-020-05431-y

**Published:** 2020-04-11

**Authors:** Jamie M. Alberga, Anke Korfage, Ilse Bonnema, Max J. H. Witjes, Arjan Vissink, Gerry M. Raghoebar

**Affiliations:** Department of Oral and Maxillofacial Surgery, University of Groningen, University Medical Center Groningen, Groningen, the Netherlands

**Keywords:** Dental implants, Head and neck cancer, Tooth extraction, Radiation therapy, Patient satisfaction

## Abstract

**Background:**

Little is known about immediate implant placement in head and neck cancer patients. We studied implant survival and functional outcomes of overdentures fabricated on implants placed immediately after removal of the lower dentition during ablative surgery or preceding primary radiotherapy (RT).

**Methods:**

Inclusion criteria were primary head and neck cancer, dentate lower jaw, and indication for removal of remaining teeth. Two implants to support a mandibular overdenture were placed immediately after extraction of the dentition during ablative surgery, or prior to starting primary radiotherapy. Standardized questionnaires and clinical assessments were conducted (median follow-up 18.5 months, IQR 13.3).

**Results:**

Fifty-eight implants were placed in 29 patients. Four implants were lost (implant survival rate 93.1%). In 9 patients, no functional overdenture could be made. All patients were satisfied with their dentures.

**Conclusions:**

Combining dental implant placement with removal of remaining teeth preceding head neck oncology treatment results in a favorable treatment outcome.

**Electronic supplementary material:**

The online version of this article (10.1007/s00520-020-05431-y) contains supplementary material, which is available to authorized users.

## Introduction

In patients with malignancies in the oral cavity, oral foci (caries profunda, periodontal disease, presence of periapical pathology) are frequently encountered during pre-radiation dental screening [1–3]. Detection and elimination of these oral foci before starting treatment are needed for patients in need of radiation therapy to prevent post-radiation oral sequelae. For some patients, elimination of oral foci implies removal of all remaining teeth during tumor resection. In patients who will undergo primary radiotherapy, the teeth are usually removed 2–3 weeks before starting radiotherapy [4]. Patients are often left with a strongly reduced oral function due to the changed anatomical situation after surgery. When ablative surgery is followed by radiotherapy, oral function is additionally compromised due to reduced salivary secretion, reduced chewing, swallowing, and radiotherapy-induced trismus.

Fabricating a functional conventional denture in the lower jaw is challenging and sometimes even impossible [5, 6]. Dental implant placement in patients with oral cancer results in improvement of oral function after oncological treatment [7–9]. Lower implant survival rates have been associated with radiotherapy; however, with appropriate perioperative measurements and strict monitoring, irradiated patients can also benefit from dental implant placement [10–15].

Several options exist regarding timing of implant placement in oncology patients. Implants can be inserted after oncologic treatment is completed. When necessary, extraction of the remaining dentition is carried out during ablative surgery, and implants are placed in a second surgery when the surgical defect is fully healed and patients have completed postoperative radiotherapy. A possible benefit of this method is that proper implant planning and positioning can be achieved to facilitate the implant supported overdenture. Also, when postponing the decision to start implant treatment until after oncologic surgery or radiotherapy, the clinician has the opportunity to only select those patients with severe functional problems who are presumed to benefit from an implant-supported overdenture. In this manner, no implants are placed in patient who will not use them or who will be deceased before starting the prosthetic rehabilitation process. This could be of value from a cost-effectiveness point of view. A major disadvantage is the need for additional surgical procedures. Oral cancer patients have shown to decline additional implant surgery after finishing the oncologic treatment, even when significant benefits were to be gained from the treatment. This issue is probably related to treatment exhaustion [16]. Another disadvantage when treating patients after oncologic therapy is that patients who have received radiotherapy may need antibiotic prophylaxis and/or a course of pre-treatment hyperbaric oxygen therapy in order to prevent osteoradionecrosis.

Alternatively, implants can be placed during ablative surgery. Combining implant placement and tumor surgery has certain benefits: implants are not placed in irradiated bone, patients do not need additional surgery with antibiotics or long-term hyperbaric oxygen therapy, and oral rehabilitation starts earlier, resulting in an increased quality of life [17]. Possible disadvantages of implant insertion during ablative surgery are improper implant positioning especially in patients with large defects, difficulties in acquiring sufficient keratinized mucosa around the implants, not using placed implants due to tumor recurrence, and patients refusing abutment connection surgery. In a longitudinal, prospective clinical trial on implant placement during ablative surgery refusal of abutment, connection surgery occurred in 3 out of 50 included patients [17].

Current research on implant insertion during ablative surgery has mainly focused on patients who were edentulous at the time of diagnosis or had their teeth extracted before tumor surgery in a separate procedure [8, 18–21]. These patients show high overall implant survival rates (> 90%) and are generally satisfied with the function of their implant supported overdenture [8, 18–22]. For patients with a remaining dentition which needs to be removed, there are three options regarding dental rehabilitation: (1) removal of the dentition and fabrication of a conventional denture, (2) removal of the dentition during ablative surgery followed by delayed dental implant placement in healed sites, or (3) immediate implant placement after tooth extraction in fresh extraction sockets (Supplementary Fig. 1). In healthy patients, a systematic review showed that implants placed immediately after tooth extraction are accompanied by a survival rate comparable to implants placement in healed sites [23, 24]. Even though there are studies claiming that immediate implant placement leads to a decrease in survival rates [25], the use of immediate implant therapy in specific populations, as in this study, requires consideration because of the potential benefits like a decrease in prosthetic rehabilitation time and fewer surgical procedures, to be gained from the therapy. In addition, dental loss has a negative impact on patients’ quality of life which further emphasizes the importance of adequate dental rehabilitation [26–28]. Therefore, the aim of this study was to assess performance of implants placed immediately after teeth extraction in the mandible during ablative surgery or preceding primary radiotherapy. Also, the study aims to describe oral function and denture satisfaction after immediate implant placement in patients with head and neck cancer.

## Material and methods

### Patients

All consecutive patients with a malignancy in the head and neck region referred to the head and neck center of the University Medical Center Groningen between 2014 and 2017 were screened to be included in this study. The inclusion criteria were primary tumor in the head and neck region, dentate lower jaw, and an indication for removal of the remaining dentition due to the presence of dental foci.

The oncologic treatment consisted of ablative surgery (when needed followed by postoperative radiotherapy) or primary radiotherapy (RT). Patients eligible for surgical removal of the tumor had their teeth removed during ablative surgery. These patients were offered either primary immediate mandibular implant placement (during ablative surgery), delayed mandibular implant placement (after completion of oncologic treatment), or no implant placement. For patients planned to receive primary radiotherapy, extraction of the remaining dentition followed by immediate mandibular implant placement at least 2 weeks before starting radiotherapy, delayed implant placement after radiotherapy or no implant placement was offered. All patients preferred primary immediate implant mandibular placement and informed consent was obtained. It was concluded by the Medical Ethical committee of the University Medical Center Groningen that this study was not subject to the Medical Research Involving Human Subjects Act (Number M19.234574).

After extraction of the remaining dentition, the extraction sockets were thoroughly cleaned, the height of the lower alveolar ridge was reduced, and care was taken to round off the sharp bone edges. The implant regions were prepared, and implants were placed in the native bone during ablative surgery with good primary stability of 45Ncm (Supplementary Fig. 1). Two dental implants (Brånemark Mk III TiUnite RP, Nobel Biocare, Gothenburg, Sweden) were placed in the interforaminal area in a two-stage procedure by the same surgeon (GMR). All implants were placed with antibiotic prophylaxis (amoxicillin/clavulanic acid 1000/200 mg i.v.). An osseointegration period of 3 months was considered for patients receiving only surgery and patients receiving only radiotherapy. In patients subjected to surgery followed by postoperative radiotherapy (starting 6 weeks after surgery), abutment connection surgery was postponed until after finishing the radiotherapy treatment, and the short-term side effects of radiotherapy had subsided. Abutment connection surgery was carried out under local anesthesia. Two weeks after abutment connection surgery, prosthodontic rehabilitation was started. An implant-supported overdenture was made by a maxillofacial prosthodontist. The mandibular overdentures were supported by a bar-clip construction. For all patients receiving radiotherapy, the cumulative dose at the implant locations was attained from the radiation plan provided by the radiotherapist.

### Clinical assessments

All patients were on a standardized recall schedule. After placement of the overdenture, patients were examined half-yearly by a prosthodontist. Clinical parameters, implant loss, and postoperative complications (inflammation, wound dehiscence, sequestration) were prospectively collected from the time of implant placement until final assessment in 2018.

During intraoral examination, the following clinical parameters were assessed:

- Plaque index assessed at four sites per implant (mesial, buccal, distal, lingual) using the modified plaque index [29].
- Bleeding index: assessed at four sites per implant (mesial, buccal, distal, and lingual) using the modified sulcus bleeding index [29].
- Gingiva index: Measured on a 4-point scale from 0 to 3: 0 = no visible inflammation, 1 = mild inflammation (moderate redness, mild swelling), 2 = moderate inflammation (moderate redness), 3 = severe inflammation (severe redness, swelling ulceration) [30].
- Probing pocket depth: measured to the nearest 1 mm using a manual periodontal probe (Williams Color-Coded Probe; Hu-Friedy, Chicago, IL USA) at the mesial, buccal, distal, and lingual aspects of the implants. Subsequently, the largest pocket depth for each implant was included for analysis.

### Radiographic analysis

At least 2 panoramic radiographs of each patient were made, one directly after implant insertion and one during final assessment. The change in marginal bone loss (in millimeters) in relation to the implant shoulder was calculated (Supplementary Fig. 1).

### Oral health impact, functional assessment, and patient satisfaction

Oral function and patient satisfaction were assessed when the denture had been in situ for a period of at least 6 months. Overall patient satisfaction was expressed on a 10-point rating scale ranging from very dissatisfied (1) to very satisfied (10). Denture satisfaction was specifically measured by a validated questionnaire on denture satisfaction consisting of 8 items focusing on upper and lower dentures, and on specific features such as esthetics, retention, and functional comfort [31]. Answers are given on a 5-point rating scale ranging from very satisfied (0) to very dissatisfied (4). Regarding the oral function, patients were asked to fill in a 9-item questionnaire on their ability to chew different kinds of food [32]. The Oral Health Impact Profile in short-form (OHIP-14) was used to assess the physical, psychological, and social impact of oral disease [33]. Patients were asked to answer questions about the frequency of pain, functional limitations, psychological discomfort, and social disability. Responses are made on a 5-point scale coded from never (0) to very often (4).

### Data analysis

Data analysis was performed using the Statistical Package for Social Sciences (version 23, SPSS Inc., Chicago, IL, USA). Depending on the distribution of the data, results are either expressed as mean ± standard deviation (s.d.) or median (interquartile range; IQR). When comparing data of ratio level between radiated and irradiated patients, the independent-samples *t* test was used. Between-group comparisons for ordinal data were calculated with the Mann–Whitney *U* test. *p* values less than 0.05 were considered statistically significant.

## Results

### Patients and implants

Twenty-nine patients, 15 men and 14 women, participated in this study (mean age 63.4 ± 11.1 years; range 31–81 years). Patient demographics and treatment intervals of irradiated and non-irradiated patients are presented in Tables 1 and 2. 79.3% of the patients smoked at the time of the intake. The reasons for removal of the dentition were severe periodontal disease, non-restorable caries profunda, and periapical infections.

**Table 1:** Patient characteristics regarding age at implant placement, gender, diagnosis, and type of reconstruction of the soft tissues and bone defect

Patient	Age at implant placement	Gender	Tumor	Stage	Type of reconstruction
1	65	Male	Maxillary sinus	T3N0	Primary closure
2	81	Female	Tongue	T1N1	Split thickness skin graft
3	62	Male	Oropharynx	T3N2c	N/A (primary RT)
4	61	Male	Mandibular gingiva	T4N2b	Free vascularized flap
5	63	Male	Oropharynx	T4aN2b	N/A (primary RT)
6	73	Female	Buccal mucosa	T2N0	Free vascularized flap
7	31	Male	Lower lip	T2N0	Free vascularized flap (after multiple re-excisions)
8	51	Male	Supraglottic larynx	T4N3	N/A (primary RT)
9	66	Female	Floor of the mouth	T4N1	Split thickness skin graft
10	76	Female	Mandibular gingiva	T1N0	Primary closure
11	57	Female	Mandibular gingiva	T4N0	Regional flap
12	56	Male	Oropharynx	T4apN3	N/A (primary RT)
13	62	Female	Floor of the mouth	T4N2c	Split thickness skin graft
14	71	Female	Floor of the mouth	T1N0	Local flap
15	47	Male	Mandibular gingiva	T4aN0	Free vascularized flap
16	58	Male	Oropharynx	T2cN2b	N/A (primary RT)
17	74	Female	Mandibular gingiva	T4N0	Free vascularized flap
18	62	Female	Tongue	T2N0	Primary closure
19	56	Male	Tongue	T3N2c	Split thickness skin graft
20	66	Male	Floor of the mouth	T1N0	Split thickness skin graft
21	80	Male	Supraglottic larynx	T3cN2b	N/A (primary RT)
22	69	Female	Mandibular gingiva	T1N0	Regional flap
23	49	Female	Oropharynx	T4N2c	N/A (primary RT)
24	59	Male	Floor of the mouth	T1N0	Local flap
25	60	Female	Carcinoma ex pleiomorphic adenoma of the submandibular gland	T3N0	N/A (removal of the gland)
26	71	Male	Floor of the mouth	T1N0	Split thickness skin graft
27	60	Female	Maxilla	T4N0	Free vascularized flap
28	83	Male	Tongue	T1N0	Primary closure
29	68	Female	Oropharynx	T4bN2c	N/A (primary RT)

*N*/*A*, not applicable; *RT*, radiotherapy

**Table 2:** Treatment intervals of irradiated and non-irradiated patients (months)

	Irradiated Mean (s.d.)	Non-irradiated Mean (s.d.)
Time between implant placement and second stage surgery	5.5 (2.7)	3.9 (1.3)
Time between implant placement and prosthesis placement	8.3 (3.1)	7.4 (3.9)
Time between implant placement and data collection	16.3 (9.4)	21.7 (8.9)

Eight patients (27.6%) were subjected to primary radiotherapy with a dose of 70 Gy at the tumor site. The average radiation dose at the implant site for these patients was 32.9 ± 4.8 Gy (range 27–40Gy). Thirteen patients (65.5%) were treated with postoperative radiotherapy with a mean radiation dose at the tumor site of 62.4 ± 7.4 Gy (range 46–70 Gy) and 41.1 ± 21.5 Gy (range 2.1–64.6 Gy) at the implant region. Eight patients were treated by surgery only. One patient treated with postoperative radiotherapy developed osteoradionecrosis near the implant region which healed after a sequestrectomy under local anesthesia.

During the first 2 weeks postoperatively, there were no problems with wound healing related to the implant procedure. Four implants in three patients were lost during follow-up which results in an overall implant survival rate of 93.1%. Implant loss was not associated with smoking. The implants that were lost had been in situ for a mean period of 17.3 ± 15.4 months (range 7–35 months). All implant losses occurred in irradiated patients (primary RT *n* = 2; postoperative RT *n* = 1) who received a radiation dose above 40 Gy at the implant site. This leads to implant survival rates of 90.5% and 100%, respectively, in irradiated and non-irradiated patients. The primary tumor in the patients with implant loss was located in the oropharynx (*n* = 2; T2-T3 tumors) or floor of mouth (*n* = 1; T4 tumor). One patient received a new implant 3 months after the old implant was removed. The new implant was placed under local anesthesia with antibiotic prophylaxis. The second patient lost both implants and continued to wear a conventional denture. The third patient lost one implant and continued to wear an implant-supported overdenture.

In 9 patients, no functional implant-retained overdenture could be made because of tumor recurrence in the implant region or metastatic tumor growth (*n* = 5), implant loss (*n* = 2), or severe pain in the implant area (*n* = 1). One patient did not show up for further follow-up (Supplementary Fig. 2). The remaining 20 patients received implant-retained mandibular overdentures of which 13 had undergone radiotherapy. After overdenture placement, seven patients had to be excluded for further assessment, due to refusing further follow-up at the prosthodontist and oncologic surgeon after receiving the overdenture (*n* = 3), tumor recurrence or death (*n* = 2), dehiscence occurrence around the reconstruction plate in such manner that the patient was not allowed to wear the fabricated overdenture (*n* = 1), and implant loss (*n* = 1). Ultimately, 13 patients received oral function questionnaires. Of these 13 patients, 8 patients had received radiotherapy.

### Clinical and radiographic analysis

The plaque and bleeding scores around the implants were considered low for all patients in the study group. Mean probing pocket depth was 2.3 ± 0.4 mm with a mean marginal bone loss around the implants of 1 ± 0.7 mm. Peri-implant bone loss was greater (but not statistically significant) in irradiated patients than in patients who were treated by surgery only (respectively, 1.5 mm and 0.9 mm). There were no clinically and statistically significant differences between irradiated and non-irradiated patient with the exception of the bleeding index (Table 3).

**Table 3:** Results of periodontal indices around the implants between irradiated patients and non-irradiated patients

	Surgery and postoperative RT			Only surgery			
	Median	Mean	s.d.	Median	Mean	s.d.	*p* value
Bleeding index (0–3)	0	0.4	0.7	1	1.2	0.8	0.04
Plaque index (0–3)	1	0.8	0.7	1	0.8	0.4	0.80
Pocket depth (mm)	NA	2.2	0.4	NA	2.5	0.3	0.18
Marginal bone loss (mm)	NA	2	0.6	NA	1	0.9	0.17

### Oral health impact, functional assessment, and patient satisfaction

Results of the OHIP-14, total chewing ability, and denture satisfaction are presented in Table 4. A higher score for denture satisfaction and chew function indicates a less satisfied patient and worse chew function. A higher OHIP-14 score indicates a higher physical, psychological, and social impact of the oral disease. The results show reasonably satisfied patients and good oral function for all three types of food. No statistically significant differences could be found in chewing ability and satisfaction rates between irradiated and non-irradiated patients (Table 5).

**Table 4:** Patient satisfaction, functional assessment and oral health impact

	Mean (s.d.)
Overall satisfaction [0–10]*	8.6 (0.9)
Total denture satisfaction score [8–40]	12.6 (3.6)
Chew function score [0–18]^a^	
Soft food	0.0 (0.1)
Tough food	0.2 (0.3)
Hard food	0.9 (0.9)
OHIP-14 total	5.8 (5.7)

*Range 0–10: 0 = very dissatisfied, 10 = very satisfied

^a^Range 0–2: scale 0 = good, 1 = moderate, 2 = bad

**Table 5:** Differences in oral function between patients with and without radiotherapy

	Irradiated Mean (s.d.)	Non-irradiated Mean (s.d.)	*p* value
Overall satisfaction [0–10]	9 (0.9)	8 (0.7)	0.08
Denture satisfaction [8–40]	10.8 (3.4)	14.8 (2.8)	0.10
Chew function [0–18]	3.2 (3.0)	3.2 (3.9)	0.93
OHIP-14 total	3.8 (3.8)	8.2 (6.9)	0.27

## Discussion

This study aimed to assess the treatment outcomes of mandibular implants placed immediately after removal of the dentition in head and neck cancer patients. The results showed a high implant survival rate for non-irradiated patients and a reduced survival rate in irradiated patients. The implant survival rates are comparable to the edentulous patients in previous studies [17, 19, 20].

A history of radiation therapy is not considered a contraindication for implant placement as long as strict monitoring is provided to prevent complications [12]. Previous studies on implant placement in irradiated patients do not regard immediate implant placement, making a comparison between our study and previously published studies not entirely reliable [10–15]. But as all implant losses in our study occurred in irradiated patients, it can be stated that radiotherapy also has a negative effect on survival of immediately placed implants. Due to the small sample size in the current study, no reliable conclusion could be drawn on implant survival rates or the proportion of unused implants in relation to tumor stage or tumor location. The optimal time between implant placement and start of radiotherapy is still in need of further research. One could argue that the osseointegration of implants placed pre-radiation therapy has already largely taken place before the bone is compromised by radiotherapy [34], but it is known that late effects of radiotherapy continue years after the initial treatment is finished. The implants in our study were on average inserted 5.3 weeks before starting postoperative radiotherapy and 2.9 weeks before starting primary radiotherapy. Thus, the implants were not in the process of osseointegration when radiotherapy was started, and this could have played a role in the implant loss in the irradiated patients. It is, however, from an oncologic treatment perspective, not preferable to further delay the start of radiation therapy. All irradiated patients in the current study received intensity-modulated radiation therapy (IMRT). A tendency towards more bone loss in irradiated patients than in those without radiotherapy was seen. This finding is comparable to the findings of Ernst et al. [35]. In a recent study of Papi et al. [36], the type of radiotherapy (3D conform radiotherapy versus IMRT) does not seem to effect the amount of peri-implant bone loss.

One of the advantages of primary implant placement is the early prosthodontic rehabilitation in head and neck oncology patients as confirmed by the studies of Wetzels et al. [8] and Mizbah et al. [21]. In an earlier study of Korfage et al. [37], non-irradiated patients received their overdenture after 6 months, and irradiated patients received their overdenture after 11 months. This difference in loading time is due to the minimal time-span of 6 months applied for irradiated patients between the end of radiotherapy and abutment connection surgery. The rationale behind this additional healing period is that implants are given some extra time for osseointegration and that the radiation effects on the soft-tissue will have subsided. Schoen et al. [16] have already questioned whether the additional 6 months is really necessary because most of the osseointegration takes place during the first 6 weeks after placement. In our study, abutment connection surgery in irradiated patients took place as soon as the treatments at the department of radiotherapy were completed and the short-term side effects of radiotherapy had subsided. This probably resulted in a shortening of the time until overdenture placement (8.3 months).

A possible disadvantage of implant placement during ablative surgery is improper positioning of implants, due to an altered anatomical situation or intermaxillary relationship, e.g., after mandibular continuity resections. In our study, all primary implants could be placed in a proper position, even in patients with a tumor located more ventral in the floor of the mouth. In those cases, it is often difficult to acquire enough keratinized mucosa around the implants and sometimes a secondary mucosa graft might be necessary. This was not needed in the current study.

Satisfaction rates and oral function do not seem to be influenced by radiotherapy in the current study. Overall, it could be stated that the results are comparable to previously edentulous patients [20]. The results in our study are to be considered as short-term results but it is expected that the oral function will be rather stable, as this is also the case in an earlier study from our group [7]. From the results of the OHIP-14 questionnaire (Table 5), non-irradiated patients seem to experience more physical, psychological, and social impact of their oral functioning than the irradiated patients. This is striking because irradiated patients are expected to experience a larger oral health-related impact due to the effects of radiotherapy. When examining the individual results of the patients, it was revealed that the higher OHIP-14 score in non-irradiated patients in our study is caused by one patient being particularly dissatisfied, therefore causing a distortion in the results because of the low number of included patients.

Another known disadvantage of implant placement during ablative surgery is the risk of unused implants due to tumor recurrence. In our study, this was the case for 5 patients. Four of these patients presented with large (T4 stage) tumors with regional metastases in combination with other comorbidities like diabetes mellitus, COPD, and hypertension. The other patient had multiple tumor recurrences which resulted in the implants eventually not being used. Furthermore, 4 patients (13.7%) refused further follow-up, 3 of these 4 patients after having received the implant-supported denture. These patients also declined further oncological follow-up despite efforts with regard to the need for regular follow-up. Numbers on head and neck cancer patients declining follow-up for their implant-supported overdenture are unknown, but a study by Toljanic et al. [38] on dental follow-up of irradiated head and neck cancer patients stated that dental follow-up compliance is an issue in this population.

The question raises whether placing implants prior to radiotherapy or during ablative surgery is cost-effective. In the Netherlands, the costs of implant rehabilitation in head and neck cancer patients are covered by the insurance which makes primary immediate implant placement an actual treatment option. A study of Wetzels et al. [39] on cost-effectiveness stated that individual costs of implant placement during ablative surgery have shown to be lower when compared to postponed placement, but that factors as oncological prognosis and overall life expectancy must be taken into account when considering placing implants during ablative surgery. The same conditions should apply for immediate implant placement.

Although this is the first study to report solely on immediate implant placement in head and neck cancer patients, there are certain limitations. A drawback is the low number of patients and the rather high fall-out rate. Regarding the method of measuring the marginal bone loss, it would be preferable to use standardized intra-oral dental radiographs. However, clinical experience with oral cancer teaches that in anatomically altered patients, there is often no possibility for taking standardized intra-oral radiographs with individual devices, and panoramic radiographs are the only option for routine follow-up. Moreover, for evaluation of bone around implants, panoramic radiographs are widely used and accepted, despite that they distort images, superimpose bony structures of the spine, and lack sharpness [40]. This should be taken into account when interpreting these results. Recommendations for further research in this field of work include identifying possible predictive factors for implant survival and setting up a treatment algorithm for clinicians.

Despite the limitations of the study, immediate primary implant placement is a viable treatment option and should be offered to head and neck cancer patients because of the earlier mentioned benefits to be gained.

## Electronic supplementary material

ESM 1 (DOCX 3384 kb)
