# Supplementary material for: Mandibular dental implant placement immediately after teeth removal in head and neck cancer patients
Source: Support Care Cancer. 2020 Apr 11;28(12):5911–8. doi: 10.1007/s00520-020-05431-y (PMC7686200; doi:10.1007/s00520-020-05431-y)
Supplement: Supplementary file 1 — (DOCX 3384 kb) [file 520_2020_5431_MOESM1_ESM.docx]

*Supplementary figure 1a. Panoramic radiograph of a patient with a squamous cell carcinoma located in the floor of the mouth.*


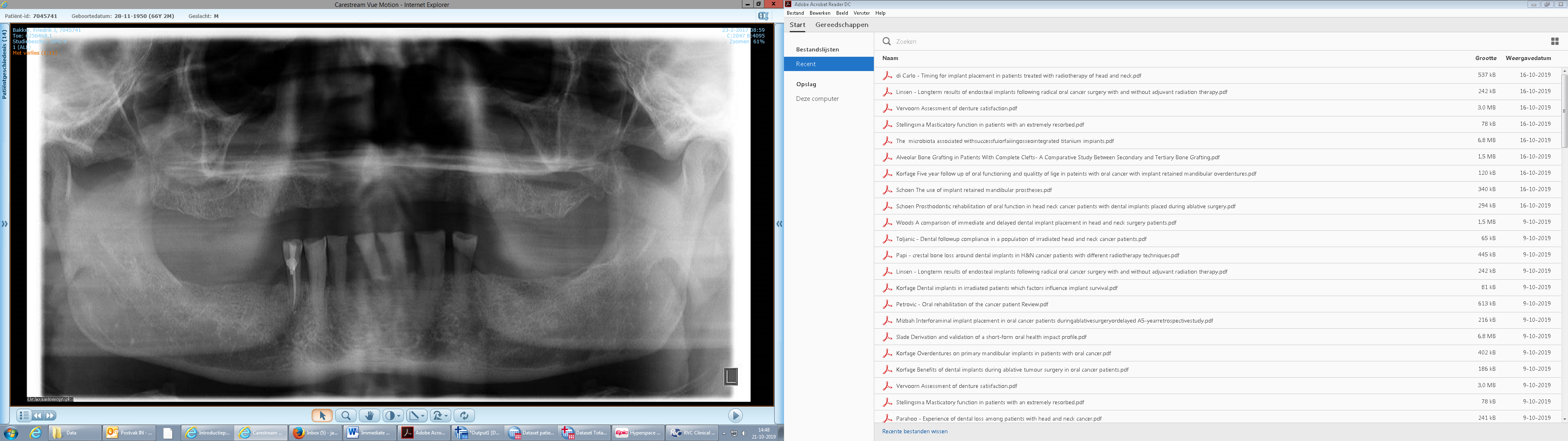


*Supplementary figure 1b. Clinical situation after preparation of the implant sites.*

*
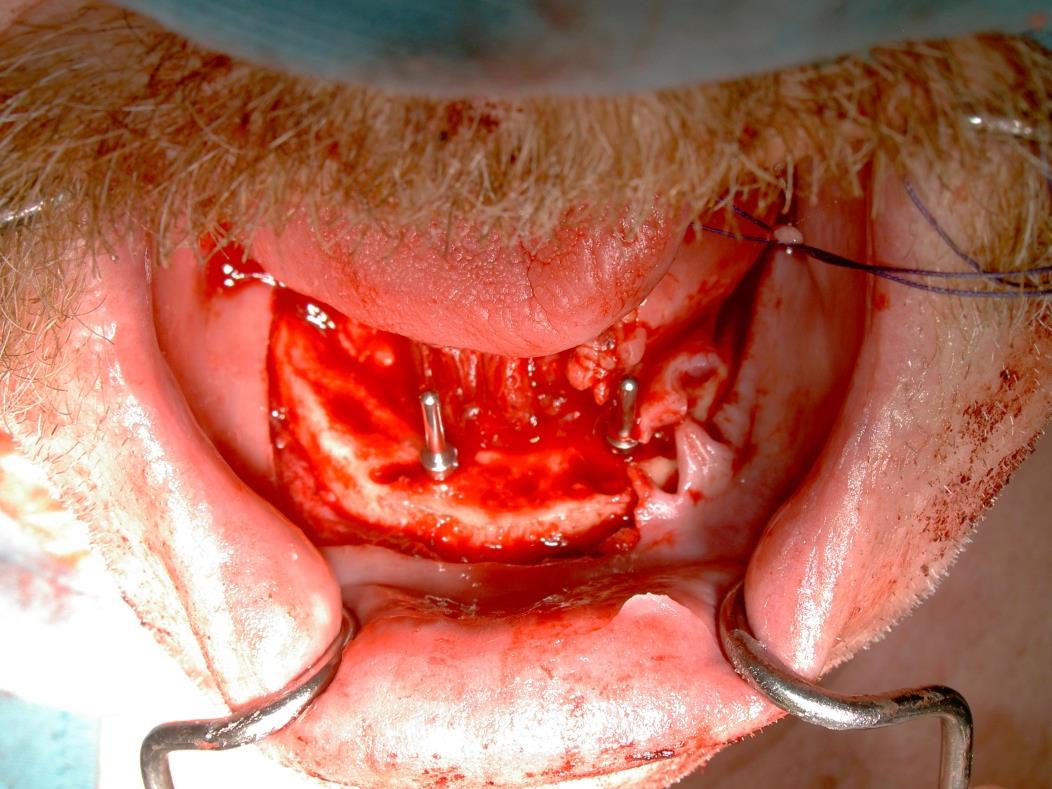
*

*Supplementary figure 1c. Panoramic radiograph immediately after teeth extraction and implant placement.*


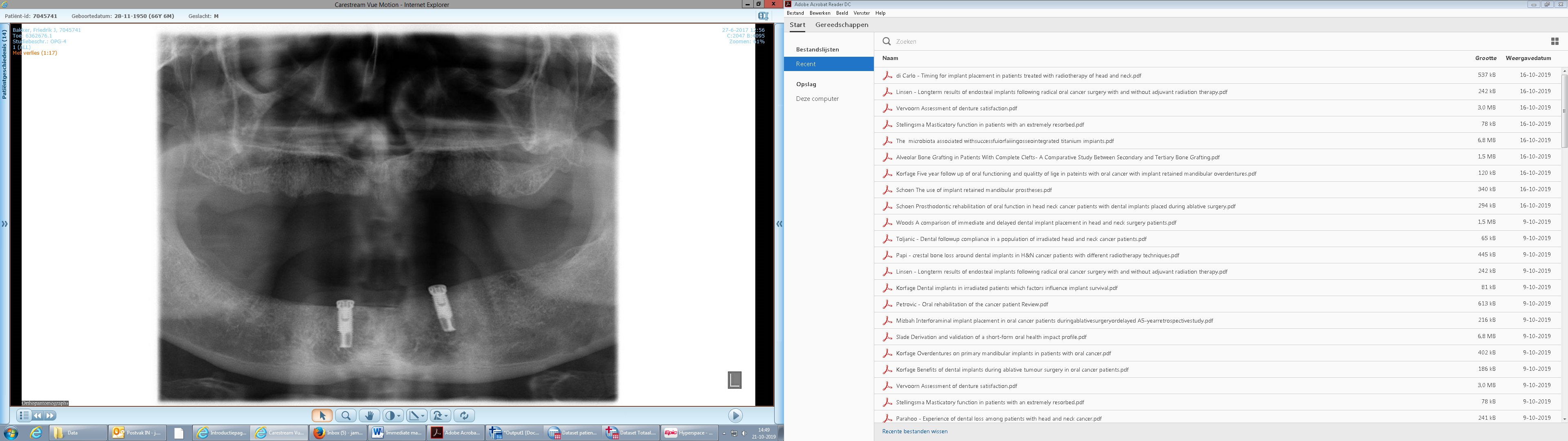


*Supplementary figure 1d. Panoramic radiograph 1.5 year after implant placement. There are no signs of peri-implant bone loss.*


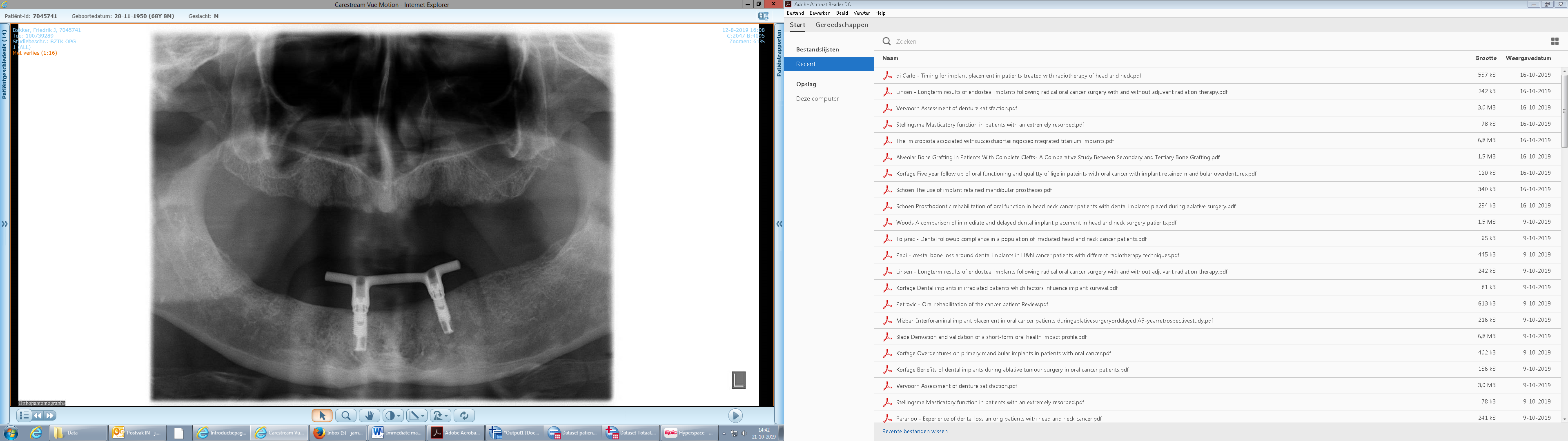
*.*

*Supplementary* *figure 2. Algorithm showing the selection of patients.*
